# Brand Cigarillos: Low Price but High Particulate Matter Levels—Is Their Favorable Taxation in the European Union Justified?

**DOI:** 10.3390/ijerph120809141

**Published:** 2015-08-06

**Authors:** Julia Wasel, Michael Boll, Michaela Schulze, Daniel Mueller, Matthias Bundschuh, David A. Groneberg, Alexander Gerber

**Affiliations:** Institute of Occupational Medicine, Social Medicine and Environmental Medicine, Goethe-University, Theodor-Stern-Kai 7, Haus 9b, Frankfurt am Main 60590, Germany; E-Mails: julia.wasel@arcor.de (J.W.); occup-med@uni-frankfurt.de (M.B.); Michaela.Schulze@stud.uni-frankfurt.de (M.S.); Daniel.mueller@med.uni-frankfurt.de (D.M); bundschuh@med.uni-frankfurt.de (M.B.); groneberg@med.uni-frankfurt.de (D.A.G.)

**Keywords:** particulate matter, PM, tobacco smoke, cigarillos, ETS

## Abstract

*Background*: Second hand smoke (ETS)-associated particulate matter (PM) contributes considerably to indoor air contamination and constitutes a health risk for passive smokers. Easy to measure, PM is a useful parameter to estimate the dosage of ETS that passive smokers are exposed to. Apart from its suitability as a surrogate parameter for ETS-exposure, PM itself affects human morbidity and mortality in a dose-dependent manner. We think that ETS-associated PM should be considered an independent hazard factor, separately from the many other known harmful compounds of ETS. We believe that brand-specific and tobacco-product-specific differences in the release of PM matter and that these differences are of public interest. *Methods*: To generate ETS of cigarettes and cigarillos as standardized and reproducible as possible, an automatic second hand smoke emitter (AETSE) was developed and placed in a glass chamber. L&M cigarettes (“without additives”, “red label”, “blue label”), L&M filtered cigarillos (“red”) and 3R4F standard research cigarettes (as reference) were smoked automatically according to a self-developed, standardized protocol until the tobacco product was smoked down to 8 mm distance from the tipping paper of the filter. *Results*: Mean concentration (Cmean) and area under the curve (AUC) in a plot of PM_2.5_ against time were measured, and compared. CmeanPM_2.5_ were found to be 518 μg/m^3^ for 3R4F cigarettes, 576 μg/m^3^ for L&M “without additives” (“red”), 448 μg/m^3^ for L&M “blue label”, 547 μg/m^3^ for L&M “red label”, and 755 μg/m^3^ for L&M filtered cigarillos (“red”). AUCPM2.5-values were 208,214 μg/m^3^·s for 3R4F reference cigarettes, 204,629 μg/m^3^·s for L&M “without additives” (“red”), 152,718 μg/m^3^·s for L&M “blue label”, 238,098 μg/m^3^·s for L&M “red label” and 796,909 μg/m^3^·s for L&M filtered cigarillos (“red”). *Conclusion*: Considering the large and significant differences in particulate matter emissions between cigarettes and cigarillos, we think that a favorable taxation of cigarillos is not justifiable.

## 1. Introduction

The brand L&M is produced by the Philip Morris Company, based in New York, NY, USA. L&M cigarettes are globally available and quite popular in the USA, Latin America, Central and Northern Europe, the Arab World and the Far East and South Asia. In 2013, L&M was ranked the third best-selling international cigarette brand outside the United States and China, with a 2013 shipment volume of 95 billion units [1].

Philip Morris has expanded its range of products by offering cigarillos listed under the same name as the established cigarette brand L&M. In the European Union, these cigarillos are considerably cheaper than cigarettes of the same brand mainly due to a favorable taxation (e.g., one packet L&M cigarettes, containing 20 cigarettes “red label”, “blue label” or “red without additives” costs 5 EUR compared to 2.20 EUR for a packet of 17 L&M filtered cigarillos). This price edge will enable brand-loyal cigarette smokers to stick to their usual brand despite rising cigarette taxes. This allows them to continue exposing their fellow humans including children to ETS of one brand up to decades. Several studies have shown that especially low-income smokers switch to cheaper brands or reduce consumption when cigarette prices rise [2,3]. Inconsistent taxation will counteract this effect. In the present study, we want to investigate whether there are significant differences in the amounts of ETS-associated particulate matter that different types of L&M cigarettes and L&M cigarillos emit. We believe that against the background of inconsistent taxation, these differences would be of public interest.

According to the United States Environmental Protection Agency (EPA) that defines, regulates and categorizes particulate matter (PM) in the National Air Quality Standard for Particulate Matter [4], PM is the term for a mixture of microscopic solid and liquid matter suspended in the air. Its sources are natural (e.g., fires, volcanic eruptions, *etc.*) or human activities (e.g., tobacco smoke, traffic, industrial processes or use of fossil fuels). Particulate matter is categorized into different sizes of the particles. All particles with aerodynamic diameters smaller than 10 μm are inhalable and categorized as PM_10_. The fraction of PM_10_ with an aerodynamic diameter larger than 2.5 μm is called “inhalable coarse particles”. All particles with an aerodynamic diameter smaller than 2.5 μm are called PM_2.5_ or “fine particles”. The fraction of particles with aerodynamic diameters smaller than 1 μm is called PM_1_. PM_2.5_ includes PM_1_, and consequently PM_10_ includes PM_1_ and PM_2.5_. The smaller the particles are, the more deeply they can be inhaled. Particles of the PM_1_ fraction can reach the gas exchange regions of the lung and eventually penetrate the bloodstream. Surface area and solubility also determine PM’s toxicity as they limit the extend of gasses and vapors that can be absorbed (e.g., carcinogenic benzopyrenes) permitting their transport into distal lung areas [5].

Apart from its suitability as a surrogate parameter for ETS-exposure, both long-term and short-term exposure to PM affects human morbidity and mortality, independently of its origin. According to several studies, these PM effects occur in various ways and are dose-dependent [6,7,8,9,10,11,12,13,14,15]. Many of the harmful effects are probably caused by oxidative stress, inflammation and DNA-damage [16,17]. All health effects (e.g., carcinogenesis, cardiovascular diseases, *etc.*) can also be attributed to cigarillo consumption. Unfortunately, the level of knowledge about health effects attributed to cigarillo consumption appears to be low. Many cigarillo smokers consider cigarillo smoke less problematic than cigarette smoke as long as they do not directly inhale the mainstream smoke [18,19]. We think that ETS-associated PM ought to be considered an independent hazard factor, separately from nicotine, tar, and the many other known harmful compounds of ETS. Anti-smoking legislation cannot be enforced in a non-public environment. However, better information about brand-specific differences in ETS-associated PM could help raise a general awareness.

## 2. Experimental Section

**Figure 1:**
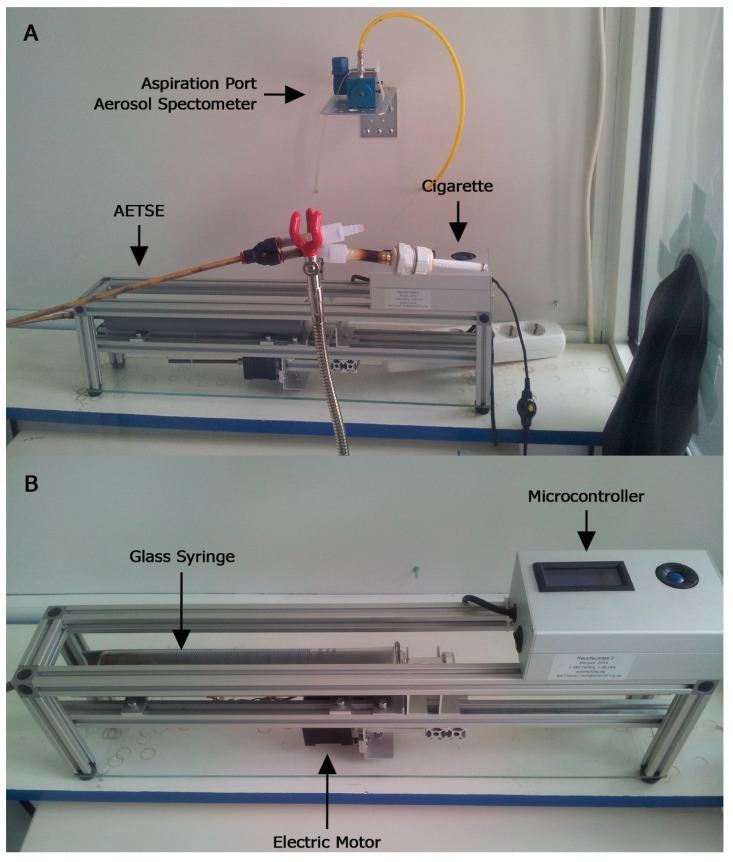
Automatic environmental tobacco smoke emitter: The AETSE consists of a glass syringe with a plunger, a stepper motor to pull and push the plunger, a micro-controller that drives the motor and aluminium parts. Two rubber gloves are embedded into the glass panel to help operate the system.

This study was performed according to the ToPIQ II study protocol [20]. Cigarettes and cigarillos were smoked in a 2.88 m^3^ glass chamber using an automatic second hand smoke emitter (AETSE, Schimpf-Ing Inc., Trondheim, Norway) (Figure 1A,B). PM_2.5_ levels were recorded, as well as temperature, air movement, and relative humidity within the chamber.

### 2.1. Tobacco Products

**Figure 2:**
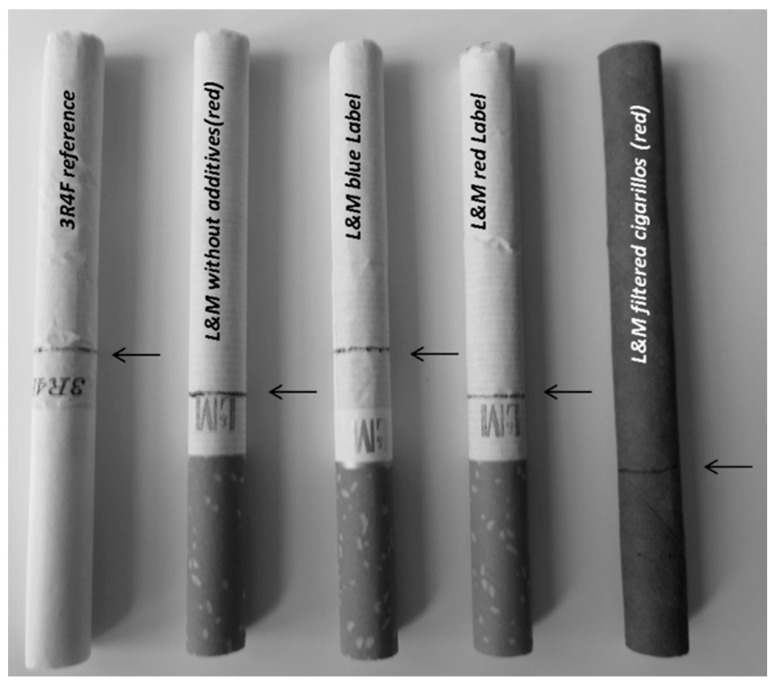
Examined tobacco products: The sample size was 20 of each tobacco product. The arrows mark a distance of 8 mm from the tipping paper. The cigarettes and cigarillos were all smoked down to this mark.

The 3R4F reference cigarette (Institute of Agriculture, University of Kentucky, USA) is made for scientific purposes only. Manufacturers’ information about 3R4F, L&M cigarettes and cigarillos are shown in Table 1. Tar, nicotine and CO amounts of L&M cigarillos are not published, as this has not been required by law. The sample size was 20 (n = 20) for each tested tobacco product (Figure 2).

L&M without additives (red), blue Label, red Label and filtered cigarillos (red) as well as standard research reference cigarette 3R4F were tested. The first six features are given on the package of the tobacco product. Further details were added from our own measurements.

**Table 1:** Features of tested tobacco products.

Features	3R4F Reference	L&M without Additive (Red)	L&M Blue	L&M Red	L&M Filtered Cigarillos (Red)
producer	Univ. of Kentucky	Philip Morris GmbH	Philip Morris GmbH	Philip Morris GmbH	Philip Morris GmbH
price [€]	0.15	5	5	5	2.20
units per package	20	20	20	20	17
tar content [mg]	9.4	10	6	10	no information
nicotine content [mg]	0.73	0.9	0.5	0.8	no information
carbon monoxide [mg]	12.0	10	7	10	no information
length [mm]	84	83	83	83	84
weight [mg]	988	787	771	828	1241
filter length [mm]	27	20	26	20	7
filter diameter [mm]	8	8	8	8	8
filter weight [mg]	170	112	147	105	35
tobacco weight [mg]	775	582	528	623	869

### 2.2. Analytical Unit/Automatic Environmental Tobacco Smoke Emitter (AETSE)

We describe the AETSE in the ToPIQ II study protocol [20]. The AETSE was developed and constructed according to our needs by the electrical engineering company Schimpf-Ing, Trondheim, Norway. Its purpose is to generate tobacco smoke in a standardized way as reliable and reproducible as possible. The AETSE consists of a 500 mL glass syringe and a linear-powered stepping motor, which imitates puffing by pulling and pushing the syringe plunger. The MS is sucked into the syringe and exhaled into the chamber Figure 1A, passing hoses and two check valves. The AETSE is triggered by a micro-controller. All parts are screwed together and fixed within a framework of aluminum profiles Figure 1B. Two rubber gloves, embedded into the side wall of the chamber, provide isolated access and enable the experimenter to ignite and extinguish the cigarettes and cigarillos without opening the door and being exposed to ETS (Figure 1A).

### 2.3. Aerosol Spectrometer

**Figure 3:**
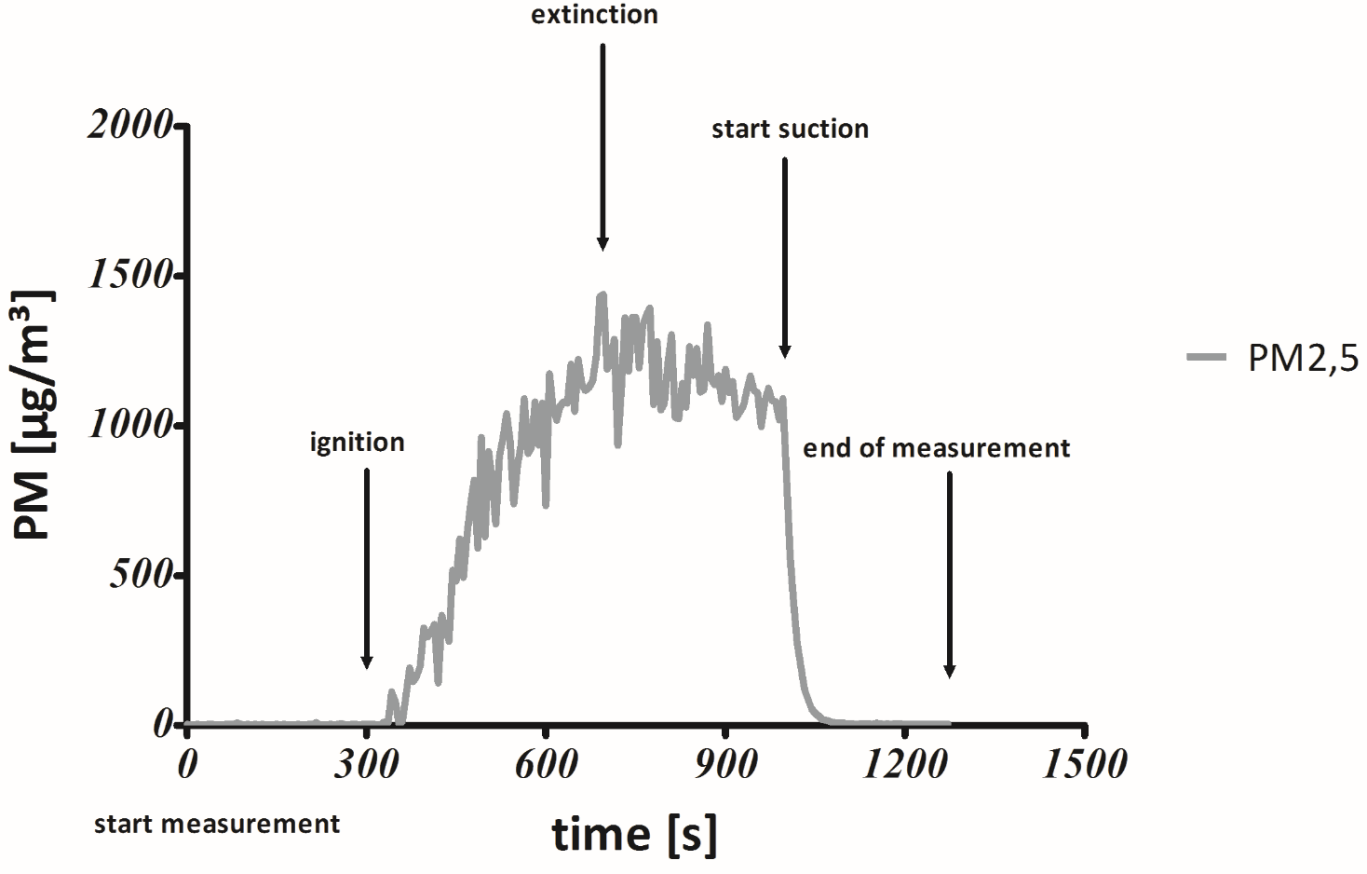
The measuring cycle: A complete measuring cycle illustrated exemplarily. The interval from ignition to extinction was evaluated.

An aerosol spectrometer (Model 11.09 Grimm Co., Ainring, Germany) is used to quantify the concentration of PM_2.5_ using a special weighting function (particles < 0.5 μmm: 100%, particles = 2.5 μm: 50%, particles > 3.5 μm: 0%). For our ETS-experiments, we used the Grimm manufacturer’s factory calibration, which was not tested for tobacco smoke. The aerosol spectrometer operates with a volume-flow-rate of 1.2 L/min. (volume controlled) and a time resolution of 6 s. To protect the damageable metrology from the sticky condensates of the ETS, the sample air was diluted pre-analytically at a ratio of 1:10, using the VKL mini (Model 7.951, Grimm Co., Ainring, Germany) and neutral compressed air. The dilution system is fixed at the back panel inside the chamber at the height of 1.70 m. The sample air is subsequently sucked from inside the chamber through the back panel via a 15 cm suction hose to the intake socket of the aerosol spectrometer, which is placed on a board at the same height at the back panel outside the chamber. The laboratory rooms were air-conditioned and kept at a temperature of 22.5 ± 2 °C and a humidity of 29% ± 5% ensuring that daily variations of environmental PM concentration not influence our measurements. Nevertheless, background PM concentrations were documented before and after each measuring cycle by reading the baseline (Figure 3).

### 2.4. Smoking Protocol

Figure 3 illustrates the sequences of our self- developed smoking protocol. Our aim was to generate tobacco smoke from cigarettes and cigarillos in a standardized way as realistic and as repeatable as possible. Unlike in the ToPIQ II study protocol, the smoking duration was not limited by 10 identical puffs. In order to take the different smoking durations of cigarettes and cigarillos into consideration, the tobacco product was extinguished as soon as it had burned down to a defined mark, which was set at a distance of 8 mm to the tipping paper Table 2. We consider 8 mm a realistic mean butt length. Puff duration was 3 s, puff volume 40 mL and inter-puff smoldering-interval 27 s. Measurement for statistical evaluation started with an initial double puff to support manual ignition of the tobacco product and ended with the extinction of the tobacco product in a water quench, when the defined mark was reached. Each measuring cycle finished with dust extraction using an industrial suction on the roof and in the back panel of the chamber.

**Table 2:** Rod length burned, duration of smoking and number of drags taken to reach the defined mark (8 mm after tipping paper).

Smoking parameters	3R4F Reference	L&M without Additives (Red)	L&M Blue	L&M Red	L&M Filtered Cigarillo (Red)
number tested (n)	20	20	20	20	20
minimum duration of smoking [s]	300	285	270	345	660
maximum duration of smoking [s]	450	400	460	520	1380
mean duration of smoking [s]	404	357	355	438	1054
mean number of drags taken	13	10	10	14	35

### 2.5. Data Processing and Analysis

**Figure 4:**
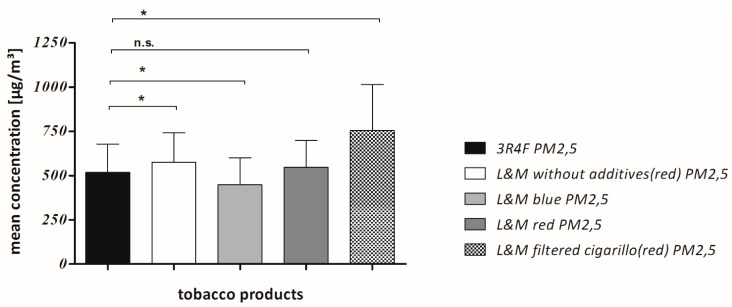
Mean concentrations are compared: The interval from ignition to extinction was evaluated for each tobacco product and mean concentrations were compared. “*” means significant (*p* < 0.05), “n.s.” means not significant.

**Figure 5:**
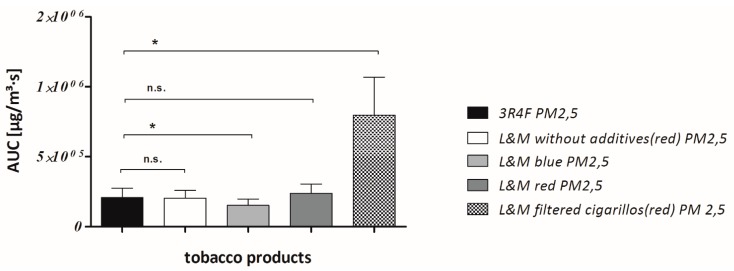
AUC are compared: The interval from ignition to extinction was evaluated for each tobacco product and AUC were compared. “*” means significant (*p* < 0.05), “n.s.” means not significant.

The statistic program “Graph Pad Prism 5.03” was used for all statistical calculations and graphic representations. Cmean PM_2.5_ was measured in the timespan between ignition and extinction of each cigarette and cigarillo Figure 3 and the area under the curve (AUC) PM_2.5_ was calculated as integral in a plot of CmeanPM_2.5_ from ignition to extinction of the tobacco product against the time. This method is commonly known from toxico-pharmacological studies were it is usually used in plots of drugs in blood plasma against time. Twenty tobacco products of each type (n = 20) were examined and all types were compared against the 3R4F reference cigarette respectively, using the one sample t-test. The individual exposure parameters (CmeanPM_2.5_ and AUC PM_2.5_) of all tobacco products were tested for Gaussian distribution before performing the t-test. They proved to be normally distributed. Using the one-sample *t*-test, significant differences between two tobacco products (3R4F reference + brand cigarette or brand cigarillo) were assumed when *p* < 0.05 Figure 4 and Figure 5. Finally, Bonferroni’s correction was performed to counteract the problem of multiple comparisons.

## 3. Results and Discussion

The results of our measurements are shown in Table 3. Statistically significant differences in Cmean PM_2.5_ were found for L&M without additives (red) cigarettes, L&M blue cigarettes and L&M cigarillos (red) compared with the 3R4F reference cigarette respectively. Regarding AUC PM_2.5_, differences were significant for L&M blue cigarettes and L&M cigarillos (red) compared with the 3R4F as a reference respectively Figure 4 and Figure 5. PM_2.5_ mean concentration of L&M cigarillos exceeded those of the 3R4F research cigarette by 69%. When comparing the AUC PM2.5 of L&M cigarillos with the 3R4F cigarette, the cigarillos emit an even 3.8 fold higher dosage than the 3R4F cigarettes. Of all tested tobacco products, L&M filtered cigarillos proved to emit highest amounts of both Cmean PM_2.5_ and AUC PM_2.5_.

**Table 3:** Cmean- and AUC-values of PM2.5 fractions.

PM2.5-Values	3R4F Reference	L&M without Additives (Red)	L&M Blue	L&M Red	L&M Filtered Cigarillo (Red)
C_mean_ PM2.5 (μg/m^3^)	518 ± 161	576 ± 166	448 ± 154	547 ± 153	755 ± 259
AUC PM2.5 (μg/m^3^·s)	208,214 ± 67,324	204,629 ± 55,191	152,718 ± 45,183	238,098 ± 67652	796,909 ± 271710

In the present study we want to juxtapose PM_2.5_ amounts generated by L&M filtered brand cigarettes and L&M filtered brand cigarillos, compared to PM_2.5_ generated by filtered 3R4F reference cigarettes, using an automatic environmental tobacco smoke emitter and an aerosol spectrometer. We would like to emphasize, however, that we were not primarily interested in the absolute amounts of Cmean and AUC PM_2.5_. All absolute data remain imprecise, as long as the aerosol spectrometer is not calibrated against ETS. The manufacturer has not done so yet. For this study we used the Grimm manufacturer’s calibration. For future studies, we plan to create a correction factor for ETS, using a gravimetric filter. Our main focus for now lies on the relative results (the statistically significant differences between the different tobacco products). We were successful to demonstrate that the amounts of ETS-associated PM_2.5_ differ significantly between most varieties of L&M cigarettes and the 3R4F research cigarette respectively, and that L&M filtered cigarillos release considerable amounts of PM excelling those of the L&M cigarettes and the 3R4F cigarettes by far.

Every tobacco product is made of a special tobacco mixture (at the discretion of the company) including additives to ensure its unique flavor. Differences in PM emissions may be due to these production details such as the composition of tobacco, density of the tobacco filling, as well as the design of the tobacco product and addition of substances in order to improve flavor and burning qualities. The amount of cellulose, which is used as a binding substance for machine-made short filler tobacco, may also contribute to the generation of PM. On the other hand, one might assume that structure and length of a filter crucially affect the amount of PM released by a smoked cigarette—and, in fact, our findings suggest a correlation between filter length and measured PM concentration of the tested tobacco products Table 1 and Table 3. The filter seems to have a greater effect on PM than tar and nicotine quantities of the tobacco product. Wertz *et al.* (2011) call attention to the effects of additives relating to PM [21]. They demonstrate an increase of toxicants, including PM, depending on the amount of additives admixed to a tobacco product by the manufacturer. As part of the present study, we compared the standard research reference cigarette 3R4F (without additives) and the L&M without additives (red) to the L&M blue Label and L&M red Label (both with additives), but did not find any differences.

Besides the fact that L&M filtered cigarillos being equipped with the smallest filter, the amount of tobacco burned is largest in the cigarillos Table 1. Hence, L&M filtered cigarillos generate by far the largest amount of PM_2.5_ not only as far as Cmean is concerned, but also regarding AUC, the latter taking the extended period of smoking into account. This is an advantage of our chosen approach, using the AUC method and assuming that smokers do not smoke their cigarettes or cigarillos for an exact given period but rather until they are burned down.

A handicap of our method is the standard deviation of about 30% regarding the amounts of CmeanPM_2.5_ between individual packs of one brand “Table 3”. Heterogeneity was lowest in L&M “red” cigarettes (27%). Mechanical non-return valves may have contributed to this weakness of our method. They had to be replaced every time a tobacco product had been smoked down due to the clogging effects caused by the MS. As mentioned above, the aerosol spectrometer has not been calibrated against tobacco smoke, which leads to inexact absolute data. For our future studies, we plan to improve the accuracy of our measured data by means of a correction factor, which has to be generated, using a gravimetric filter. We also noticed an inter-observer variability of about 10%–20% when comparing the Cmean amounts of the 3R4F research cigarette of different projects performed by different students [20,22]. However, individual parameters of all tested varieties were normally distributed and the differences between most of the L&M varieties and the research cigarettes respectively were significant. This and the large differences between all cigarettes and the filtered cigarillos support our impression that the reliability of our method is acceptable for this purpose.

One may also ask why we did not follow an internationally accepted smoking protocol to generate our data with an AETSE (e.g., the “ISO machine smoking regime” or the “Canadian intense”) [18,23]. In fact, we followed the ISO intense regime [23] in puff frequency, but used a smaller puff volume tailor-made to the technical requirements of our AETSE. We believe our protocol is reasonable when comparing with the smoking habits of real smokers [24]. No protocol is arguably able to exactly imitate human smoking behavior with all its inter- and intra-individual variations in a realistic way. All internationally accepted protocols have been heavily criticized [25] and other research groups have reconsidered and modified parameters as well [26].

It should be mentioned, though, that our aim was not to imitate real-life conditions. We did not intend to provide absolute PM data for defined situations. We rather wanted to enable a comparison of different brands and tobacco products, using a standardized smoking protocol according to our requirements, an automatic environmental tobacco smoke emitter that works as reliably as possible within our technical possibilities, and a standard research cigarette as a reference.

In recent years, many studies have been conducted to quantify ETS-associated PM_2.5_. Measurements in outdoor smoking areas of restaurants showed mean concentrations between 8.32 μg/m^3^ and 124 μg/m^3^, depending on the number and distance of smokers to the measurement device [27]. Our own observations on public railway stations showed a comparable PM burden [28]. Other studies tested concentrations in closed rooms (e.g., the passenger cabin of a car) and documented Cmean from 85 μg/m^3^ up to 3850 μg/m^3^ [29]. We believe that specific differences in the amounts of ETS-associated PM do matter, especially when considering that in addition to their inflammatory and immunologic effects, particles may also serve as transporters for low volatile carcinogenic substances such as polycyclic aromatic hydrocarbons or aromatic amines, permitting them access to distal lung areas. Vardavas *et al.* recently demonstrated that concentrations of the carcinogenic and tobacco specific 4-(methylnitrosamino)-1-(3-pyridyl)-1-butanol (NNAL) in urine correlate with concentrations of PM_2.5_ attributable to second-hand smoke [5]. The scientists therefore examined non-smoking employees in semi-open air cafés in Athens, Greece. They found that NNAL concentrations increased by 9.5%, per 10 μg/m^3^ increase in PM_2.5_.

We consider ETS-associated PM emissions as an important independent risk factor for passive smokers. While measures to reduce tar and nicotine yield of cigarettes are already legally required in most countries, efforts to reduce PM in environmental tobacco smoke should be undertaken.

## 4. Conclusions

ETS-associated particulate matter matters. Considering the relative measurement data, large differences in the emitted amounts of harmful PM can be demonstrated, when tobacco products are smoked in a standardized way. Both the tobacco brand and the kind of tobacco product have an effect on PM. We want to illuminate the impact of these differences on the burden of PM-exposure on passive smokers.
